# Supplementary material for: Phosphorylation of WDR48 by phototropins drives starch degradation to promote stomatal opening
Source: Nat Commun. 2026 Mar 6;17:3601. doi: 10.1038/s41467-026-70314-5 (PMC13096123; doi:10.1038/s41467-026-70314-5)
Supplement: Supplementary file 1 — Supplementary Information [file 41467_2026_70314_MOESM1_ESM.pdf]

Phosphorylation of WDR48 by phototropins drives starch degradation to promote stomatal opening

Shota Yamauchi, Saashia Fuji, Hiroki Ikuta, Naoyuki Sugiyama, Yutaka Kodama, Luca Distefano, Haruki Fujii, Kota Yamashita, Hinano Takase, Mika Nomoto, Yasuomi Tada, Taishi Umezawa, Kazuhiro Hotta, Diana Santelia, Ken-ichiro Shimazaki, Atsushi Takemiya

Supplementary Figures 1–12

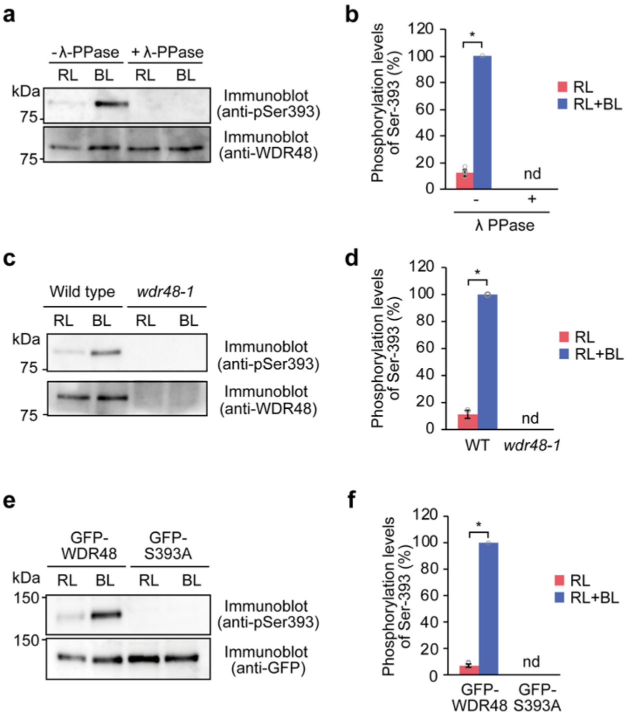

Supplementary Figure 1. Specificity of the anti-pSer393 of WDR48 antibodies.

a, b, Loss of WDR48 phosphorylation upon λ-protein phosphatase (λ-PPase) treatment. Guard cell protoplasts from the wild type were illuminated by red light (RL: 300 µmol m^{-2} s^{-1}) for 30 min, after which a pulse of blue light (BL: 100 µmol m^{-2} s^{-1}, 30 s) was superimposed on the RL. Guard cell proteins were treated with or without λ-PPase. Phosphorylation and the amount of WDR48 were detected by immunoblotting with anti-pSer393 of WDR48 and anti-WDR48 antibodies, respectively. **c–f,** Absence of WDR48 phosphorylation in the *wdr48* mutant (**c, d**) and in transgenic plants expressing the phosphodefective WDR48 mutant (**e, f**). For (**b**), (**d**), and (**f**), data represent mean ± SEM (n = 3 biologically independent samples). Asterisk indicates significant difference (Student's t-test, $P < 0.01$). nd indicates that the signal was not detected.

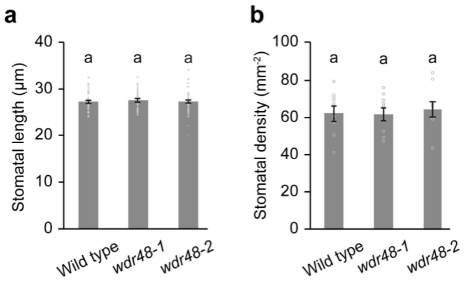

Supplementary Figure 2. Stomatal length and density in the *wdr48* mutants.

a, Stomatal length in the abaxial epidermis. Data represent mean ± SEM (*n* = 60 stomata examined over three independent experiments). **b,** Stomatal density in the abaxial epidermis. Data represent mean ± SEM (*n* = 9 biologically independent samples). The same letters indicate no significant differences (One-way ANOVA with Tukey's test, $P < 0.01$).

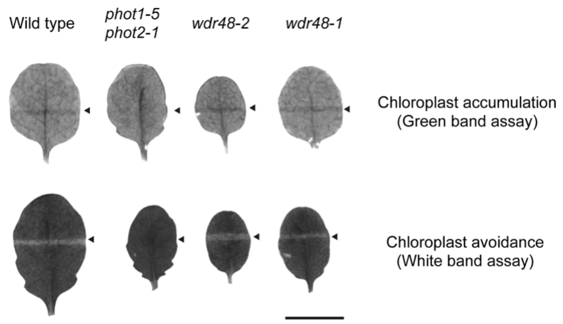

Supplementary Figure 3. Chloroplast photorelocation movements in the *wdr48* mutants.

Chloroplast accumulation and avoidance responses were detected by green and white band assays, respectively. Leaves of the wild type, *phot1 phot2*, and *wdr48* mutants were illuminated with weak blue light (0.5 µmol m^{-2} s^{-1}) or strong blue light (50 µmol m^{-2} s^{-1}) for 1 h through a slit, as indicated by arrowheads. Bar represents 1 cm.

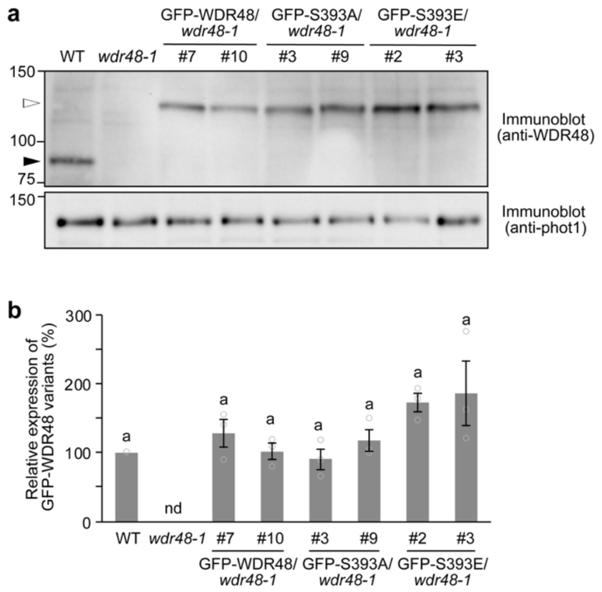

Supplementary Figure 4. Expression of GFP-WDR48, GFP-S393A, and GFP-S393E variants.
a, Immunoblot analysis using anti-WDR48 antibodies. Each lane contained 40 µg of leaf extracts. phot1 was used as a loading control. Black and white arrowheads indicate positions of endogenous WDR48 and GFP-WDR48 variants, respectively. **b,** Relative expression of GFP-WDR48 variants determined using ImageJ software. Data represent mean ± SEM (n = 3 biologically independent samples). The same letters indicate no significant differences (One-way ANOVA with Tukey's test, $P < 0.01$). nd indicates that the signal was not detected.

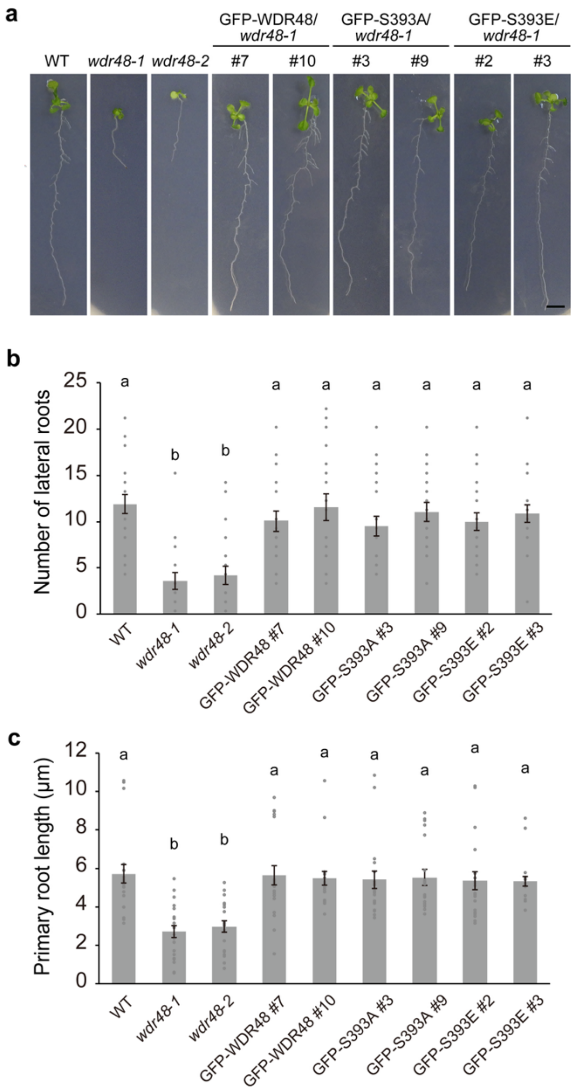

Supplementary Figure 5. Root phenotype in the *wdr48* mutants.

a, Seedlings of the wild type, *wdr48* mutants, and transgenic plants expressing phosphodefective or phosphomimetic WDR48 mutants grown for 11 days. Bar represents 1 cm. **b, c,** Quantification of lateral root number (**b**) and primary root length (**c**). Data represent mean ± SEM (n = 20 biologically independent plants; One-way ANOVA with Tukey's test, $P < 0.05$).

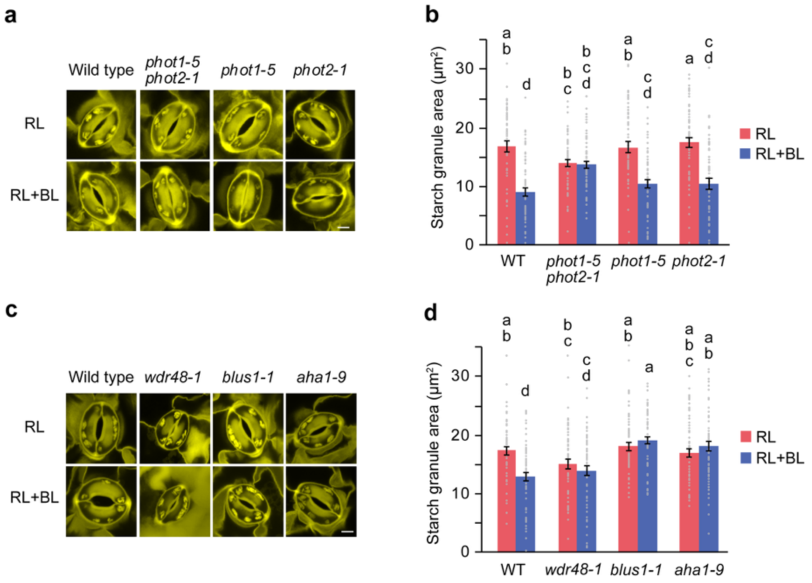

Supplementary Figure 6. Blue light-dependent starch degradation in phototropin and signalling mutants. **a–d,** Light-induced changes in starch dynamics in phototropin mutants (**a, b**) and blue-light signalling mutants (**c, d**). Epidermal stripes from dark-adapted leaves were pre-illuminated with red light (RL: 50 µmol m^{-2} s^{-1}) for 2 h, after which blue light (BL: 10 µmol m^{-2} s^{-1}) was superimposed on the background of RL for 10 min. Confocal images of PS-PI staining of the starch granules (**a, c**). Bars represent 5 µm. Quantification of starch granule areas (**b, d**). Data represent mean ± SEM (n = 60 stomata examined over three independent experiments; One-way ANOVA with Tukey's test, $P < 0.05$).

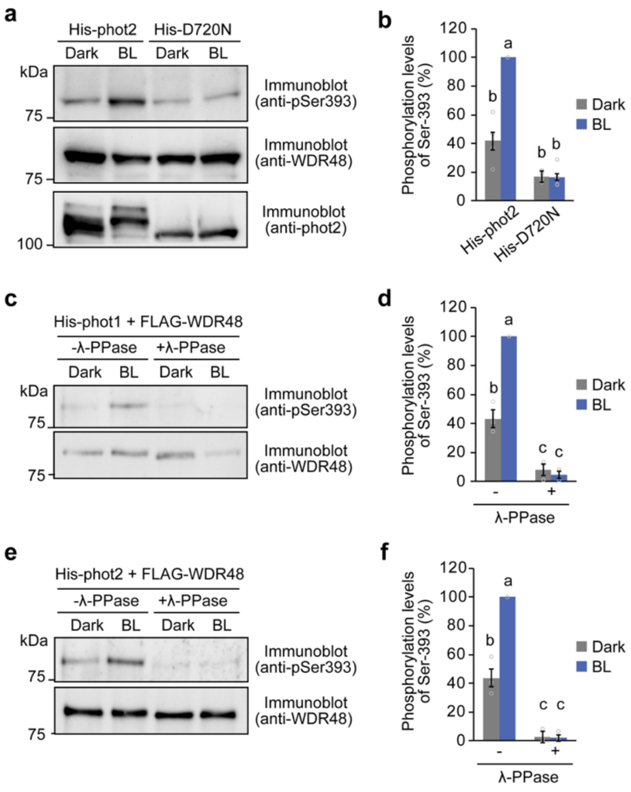

Supplementary Figure 7. *In vitro* phosphorylation of WDR48 by phot1 and phot2.

a, b, Blue light (BL)-dependent phosphorylation of WDR48 by phot2. Recombinant His-phot2 and the corresponding kinase-dead mutant of phot2 (D720N) were incubated with FLAG-WDR48 in the presence of ATP for 1 h under dark or BL conditions. Phosphorylation of WDR48 was detected by immunoblotting with anti-pSer393 of WDR48 antibodies. The amounts of WDR48 and phot2 were detected using anti-WDR48 and anti-phot2 antibodies, respectively. **c–f,** Reduction of phot1- (**c, d**) and phot2- (**e, f**) mediated WDR48 phosphorylation by λ-protein phosphatase (λ-PPase) treatment. Recombinant His-phot1 or His-phot2 was incubated with FLAG-WDR48 in the presence of ATP for 1 h under dark or BL conditions, followed by λ-phosphatase treatment. For (**b**), data represent mean ± SEM ($n = 4$ independent experiments; One-way ANOVA with Tukey's test, $P < 0.01$). For (**d**) and (**f**), data represent mean ± SEM ($n = 3$ independent experiments; One-way ANOVA with Tukey's test, $P < 0.01$).

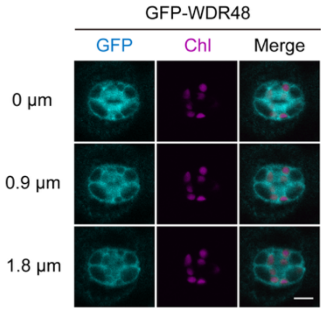

Supplementary Figure 8. Confocal images of GFP-WDR48 at different focal planes in guard cells. GFP, GFP fluorescence; Chl, chlorophyll fluorescence; Merge, merged image of GFP and Chl fluorescence. Bar represents 5 µm.

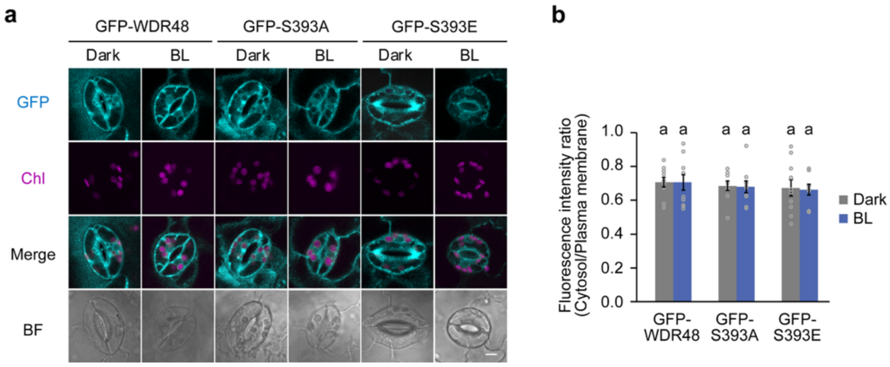

Supplementary Figure 9. Phosphodefective and phosphomimetic mutations of WDR48 do not affect the subcellular localisation of GFP-WDR48.

a, Confocal images of GFP-WDR48, GFP-S393A, and GFP-S393E in guard cells under dark and blue light (BL) conditions. GFP, GFP fluorescence; Chl, chlorophyll fluorescence; Merge, merged image of GFP and Chl fluorescence; BF, bright field. Bar represents 5 µm. **b,** The cytosol/plasma membrane fluorescence intensity ratio. Data represent mean ± SEM (*n* = 10 biologically independent cells). The same letters indicate no significant differences (One-way ANOVA with Tukey's test, $P < 0.01$).

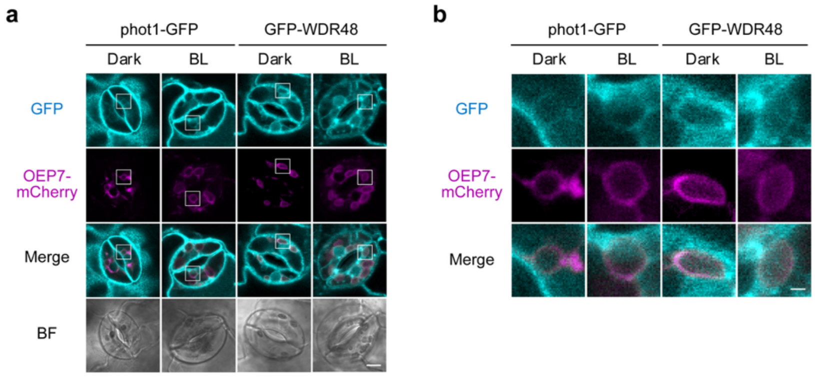

Supplementary Figure 10. Detection of phot1-GFP and GFP-WDR48 signals at the chloroplast envelope. **a, b,** Confocal images of phot1-GFP and GFP-WDR48 in guard cells under dark and blue light (BL) conditions (**a**), with the boxed region in (**a**) shown as a close-up in (**b**). Leaves from dark-adapted plants were illuminated with BL (10 µmol m^{-2} s^{-1}) for 10 min. GFP, GFP fluorescence; OEP7-mCherry, chloroplast envelope marker; Merge, merged image of GFP and OEP7-mCherry; BF, bright field. For **(a)**, bar represents 5 µm. For **(b)**, bar represents 1 µm.

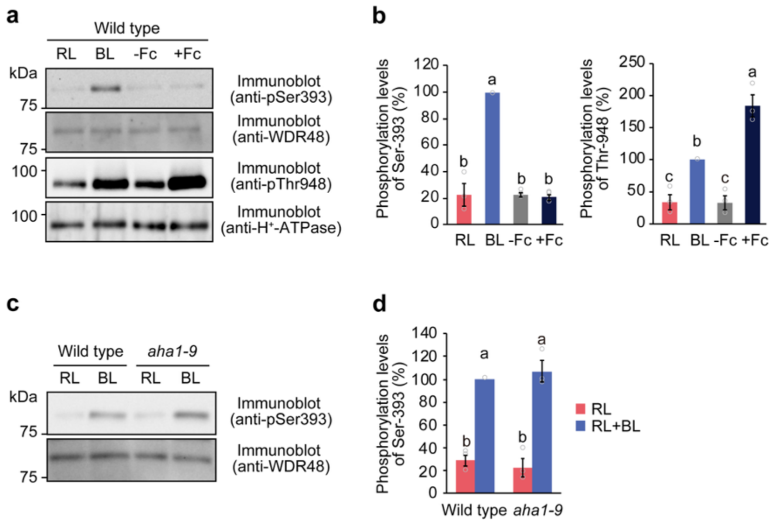

Supplementary Figure 11. WDR48 phosphorylation is independent of H^+-ATPase activation.

a, b, Blue-light (BL)- and fusicoccin (Fc)-induced changes in the phosphorylation of WDR48 and H^+-ATPase in the wild-type. Guard cell protoplasts were irradiated with red light (RL: 300 µmol m^{-2} s^{-1}) for 30 min, after which a pulse of BL (100 µmol m^{-2} s^{-1}, 30 s) was superposed on RL or 10 µM Fc was added to the protoplast suspensions. The phosphorylation and amount of WDR48 and H^+-ATPase were detected by immunoblotting with anti-pSer-393of WDR48, anti-WDR48, anti-pThr-948 of AHA1, and anti-H^+-ATPase antibodies, respectively. **c, d,** Blue light-dependent phosphorylation of WDR48 in *aha1-9* mutant guard cell protoplasts. For (**b**) and (**d**), data represent mean ± SEM (n = 3 biologically independent samples; One-way ANOVA with Tukey's test, $P < 0.05$).

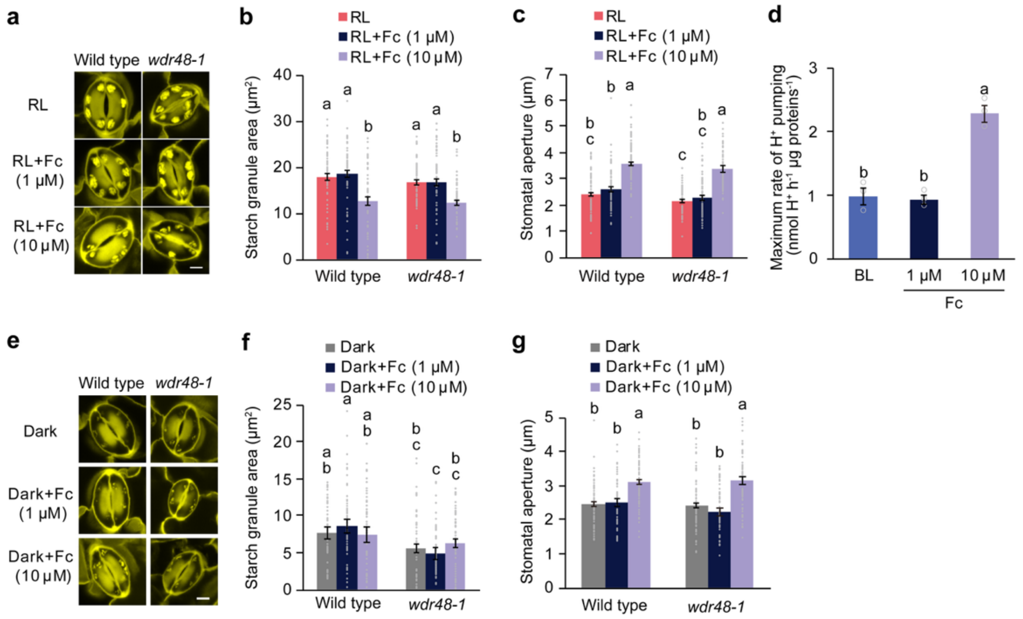

Supplementary Figure 12. Effects of high-concentration fusicoccin (Fc; 10 µM) on starch degradation, stomatal opening, and H^+ pumping.

a–c, Fc-induced changes in starch dynamics and stomatal opening under red light (RL). Confocal images of PS-PI staining of the starch granules (**a**). Epidermal stripes from dark-adapted leaves were pre-illuminated with RL (50 µmol m^{-2} s^{-1}) for 2 h, after which Fc (1 or 10 µM) was applied to the stomatal opening buffer and further incubated for 10 min under RL. Quantification of the starch granule area (**b**). Measurement of stomatal aperture (**c**). **d,** Blue light- and Fc-dependent H^+ pumping. Guard cell protoplasts were pre-illuminated with RL (300 µmol m^{-2} s^{-1}) for 2 h, after which a pulse of blue light (BL: 100 µmol m^{-2} s^{-1}, 30 s) was superimposed on the background of RL or Fc (1 or 10 µM) was applied to the protoplast suspensions. Data represent mean ± SEM (n = 3 biologically independent samples; One-way ANOVA with Tukey's test, $P < 0.05$). **e–g,** Fc-induced changes in starch dynamics and stomatal opening in the dark. Confocal images of PS-PI staining of the starch granules (**e**). Epidermal stripes from dark-adapted leaves were incubated in the dark for 2 h, after which Fc (1 or 10 µM) was applied to the stomatal opening buffer and further incubated for 10 min in the dark. Quantification of the starch granule area (**f**). Measurement of stomatal aperture (**g**). For (**a**) and (**e**), bars represent 5 µm. For (**b**) and (**f**), data represent mean ± SEM (n = 60 stomata examined over three independent experiments; One-way ANOVA with Tukey's test, $P <$ 0.05). For (**c**) and (**g**), data represent mean ± SEM (n = 75 stomata examined over three independent experiments; One-way ANOVA with Tukey's test, $P < 0.01$).
