# Supplementary material for: Phosphorylation of WDR48 by phototropins drives starch degradation to promote stomatal opening
Source: Nat Commun. 2026 Mar 6;17:3601. doi: 10.1038/s41467-026-70314-5 (PMC13096123; doi:10.1038/s41467-026-70314-5)
Supplement: Supplementary file 2 — Reporting Summary [file 41467_2026_70314_MOESM2_ESM.pdf]

nature portfolio

Corresponding author(s): Atsushi Takemiya
Last updated by author(s): Feb 19, 2026

Reporting Summary

Nature Portfolio wishes to improve the reproducibility of the work that we publish. This form provides structure for consistency and transparency in reporting. For further information on Nature Portfolio policies, see our Editorial Policies and the Editorial Policy Checklist.

Statistics

For all statistical analyses, confirm that the following items are present in the figure legend, table legend, main text, or Methods section.

n/a	Confirmed	
[ ]	[x]	The exact sample size (*n*) for each experimental group/condition, given as a discrete number and unit of measurement
[ ]	[x]	A statement on whether measurements were taken from distinct samples or whether the same sample was measured repeatedly
[ ]	[x]	The statistical test(s) used AND whether they are one- or two-sided<br>*Only common tests should be described solely by name; describe more complex techniques in the Methods section.*
[x]	[ ]	A description of all covariates tested
[x]	[ ]	A description of any assumptions or corrections, such as tests of normality and adjustment for multiple comparisons
[ ]	[x]	A full description of the statistical parameters including central tendency (e.g. means) or other basic estimates (e.g. regression coefficient) AND variation (e.g. standard deviation) or associated estimates of uncertainty (e.g. confidence intervals)
[x]	[ ]	For null hypothesis testing, the test statistic (e.g. *F*, *t*, *r*) with confidence intervals, effect sizes, degrees of freedom and *P* value noted<br>*Give P values as exact values whenever suitable.*
[x]	[ ]	For Bayesian analysis, information on the choice of priors and Markov chain Monte Carlo settings
[x]	[ ]	For hierarchical and complex designs, identification of the appropriate level for tests and full reporting of outcomes
[x]	[ ]	Estimates of effect sizes (e.g. Cohen's *d*, Pearson's *r*), indicating how they were calculated

Our web collection on statistics for biologists contains articles on many of the points above.

Software and code

Policy information about availability of computer code

Data collection	Acquisition of immunoblotting images: ChemiDoc Touch Imaging System (Bio-Rad) or FUSION-SOLO.6S.EDGE (Viber Bio Imaging)<br>Acquisition of stomata images: Eclipse TS100 (Nikon)<br>nanoLC/MS/MS: LTQ-Orbitrap (Thermo Fisher Scientific) or Orbitrap Fusion Lumos (Thermo Fisher Scientific)<br>Acquisition of confocal images: TCS SP8X (Leica Microsystems) or Digital Eclipse C1 (Nikon)<br>Gas exchange measurement: Li-6400 (Li-COR)
Data analysis	Mass Navigator v1.2 (Mitsui Knowledge Industry), Mascot version 2.3 (Matrix Science), MSFragger (version 4.1) and FragPipe platform (v22.0) were used for phosphoproteome analysis.<br>Microsoft Excel for Mac (ver. 16.104), Excel Toukei ver. 6.05 and 8.0(Esumi) were used for statistical analysis.<br>ImageJ 1.53q software (National Institutes of Health) was used for stomatal aperture measurement, quantification of signal intensity for confocal images and band intensity of immunoblotting.<br>Microsoft Excel for Mac (ver. 16.104) was used for analysis of stomatal conductance and graph drawing.<br>X-net was used for starch granule area quantification. Details are described in the Methods section.

For manuscripts utilizing custom algorithms or software that are central to the research but not yet described in published literature, software must be made available to editors and reviewers. We strongly encourage code deposition in a community repository (e.g. GitHub). See the Nature Portfolio guidelines for submitting code & software for further information.

Data

Policy information about availability of data

All manuscripts must include a data availability statement. This statement should provide the following information, where applicable:

- Accession codes, unique identifiers, or web links for publicly available datasets
- A description of any restrictions on data availability
- For clinical datasets or third party data, please ensure that the statement adheres to our policy

The data supporting finding of this study are included in the manuscript and Supplementary Information. Source data for supporting the finding is included in Source Data file.

Research involving human participants, their data, or biological material

Policy information about studies with human participants or human data. See also policy information about sex, gender (identity/presentation), and sexual orientation and race, ethnicity and racism.

Reporting on sex and gender	*Use the terms sex (biological attribute) and gender (shaped by social and cultural circumstances) carefully in order to avoid confusing both terms. Indicate if findings apply to only one sex or gender; describe whether sex and gender were considered in study design; whether sex and/or gender was determined based on self-reporting or assigned and methods used. Provide in the source data disaggregated sex and gender data, where this information has been collected, and if consent has been obtained for sharing of individual-level data; provide overall numbers in this Reporting Summary. Please state if this information has not been collected. Report sex- and gender-based analyses where performed, justify reasons for lack of sex- and gender-based analysis.*
Reporting on race, ethnicity, or other socially relevant groupings	*Please specify the socially constructed or socially relevant categorization variable(s) used in your manuscript and explain why they were used. Please note that such variables should not be used as proxies for other socially constructed/relevant variables (for example, race or ethnicity should not be used as a proxy for socioeconomic status). Provide clear definitions of the relevant terms used, how they were provided (by the participants/respondents, the researchers, or third parties), and the method(s) used to classify people into the different categories (e.g. self-report, census or administrative data, social media data, etc.) Please provide details about how you controlled for confounding variables in your analyses.*
Population characteristics	*Describe the covariate-relevant population characteristics of the human research participants (e.g. age, genotypic information, past and current diagnosis and treatment categories). If you filled out the behavioural & social sciences study design questions and have nothing to add here, write "See above."*
Recruitment	*Describe how participants were recruited. Outline any potential self-selection bias or other biases that may be present and how these are likely to impact results.*
Ethics oversight	*Identify the organization(s) that approved the study protocol.*

Note that full information on the approval of the study protocol must also be provided in the manuscript.

Field-specific reporting

Please select the one below that is the best fit for your research. If you are not sure, read the appropriate sections before making your selection.

☒ Life sciences ☐ Behavioural & social sciences ☐ Ecological, evolutionary & environmental sciences

For a reference copy of the document with all sections, see nature.com/documents/nr-reporting-summary-flat.pdf

Life sciences study design

All studies must disclose on these points even when the disclosure is negative.

Sample size	Statistical methods were not used for sample size design. Sample size for physiological and biochemical analyses was decided by referring to previous studies in our laboratory and other research groups.
Data exclusions	No data were excluded.
Replication	All experiments in this study were repeated at least three times. Number of independent experiments and replicates are available in figure legends.
Randomization	Plant materials were grown under the same laboratory condition. Samples were collected without any bias.
Blinding	Plant genotype and treatment were not blinded in this study. All conclusions regarding the results were based on statistical analysis, blinding was not performed.

Reporting for specific materials, systems and methods

We require information from authors about some types of materials, experimental systems and methods used in many studies. Here, indicate whether each material, system or method listed is relevant to your study. If you are not sure if a list item applies to your research, read the appropriate section before selecting a response.

Materials & experimental systems

n/a	Involved in the study
☐	☒ Antibodies
☒	☐ Eukaryotic cell lines
☒	☐ Palaeontology and archaeology
☒	☐ Animals and other organisms
☒	☐ Clinical data
☒	☐ Dual use research of concern
☐	☒ Plants

Methods

n/a	Involved in the study
☒	☐ ChIP-seq
☒	☐ Flow cytometry
☒	☐ MRI-based neuroimaging

Antibodies

Antibodies used

The anti-WDR48 (1:1000 dilution) and anti-pSer393 of WDR48 (1:1000 dilution) polyclonal antibodies were produced in this study. The anti-BLUS1 (1:3000 dilution), anti-pSer348 of BLUS1 (1:1000 dilution), anti-AHA1 (1:8000 dilution), and anti-pThr948 of AHA1 (1:5000 dilution) polyclonal antibodies were previously produced in our laboratory (for anti-BLUS1 and anti-pSer348 of BLUS1, Takemiya et al., 2013: for anti-H+-ATPase and anti-pThr948 of AHA1, Fuji et al., 2024). The anti-phot1 (1:3000 dilution) polyclonal antibodies were produced in previous study in our research group (Doi et al., 2004).The anti-phot2 (1:3000 dilution) polyclonal antibodies were produced in previous study in other research group (Kong et al., 2006). The anti-GFP polyclonal antibodies (1:3000 dilution) were purchased from Invitrogen (#A-6455). The monoclonal anti-FLAG M2-peroxidase (HRP) antibody (1:5000 dilution) was purchased from Sigma-Ardrich (#A8592). The anti-rabbit IgG antibody (goat) conjugated with HRP (1:3000 dilution) was purchased from Bio-rad laboratories (#1706515)

Validation

The antibodies validation and details are described in following references.

anti-WDR48 polyclonal antibodies; These antibodies were produced in this study by immunising rabbits with recombinant GST-WDR48 (V300-R753).

anti-pSer393 of WDR48 polyclonal antibodies; These antibodies were produced in this study by immunising rabbits with synthetic phospho-peptide NRARVpSLEGLNPA.

anti-H+-ATPase polyclonal antibodies; These antibodies were produced in a previous study in our laboratory by immunising rabbits with recombinant GST-AHA1 (M320-A608). Fuji, S. et al. (2024) Nat. Commun. 15, 1195

anti-pThr948 of AHA1 polyclonal antibodies; These antibodies were produced in a previous study in our laboratory by immunising rabbits with synthetic phospho-peptide IDTAGHHYpTV. Fuji, S. et al. (2024) Nat. Commun. 15, 1195

anti-BLUS1 polyclonal antibodies; These antibodies were produced in a previous study in our laboratory by immunising rabbits with recombinant GST-BLUS1 (K294-G487). Takemiya, A. et al. (2013) Nat. Commun. 4, 2094

anti-pSer348 of BLUS1 polyclonal antibodies; These antibodies were produced in a previous study in our laboratory by immunising rabbits with synthetic phospho-peptide KNRRIpSGWNF. Takemiya, A. et al. (2013) Nat. Commun. 4, 2094

anti-phot1 polyclonal antibodies; These antibodies were produced in a previous study in our research group by immunising rabbits with recombinant His-phot1 (M1-I181). Doi, M. et al. (2004) J. Exp. Bot. 55, 517–523

anti-phot2 polyclonal antibodies; These antibodies were produced in a previous study in other group by immunising rabbits with recombinant phot2 (M1-N533). Kong, S. G. et al. (2006) Plant J. 45, 994–1005

Commercial antibodies were validated and described by venders in following website
The monoclonal anti-FLAG M2-peroxidase (HRP) antibody (Sigma-Aldrich; #A8592)
https://www.sigmaaldrich.com/JP/ja/product/sigma/a8592?srsltid=AfmBOopU5YAKvpreNigtepn49DOVsi2uUqMxKxpZVPZ_GaLg7pxGGclv

The anti-rabbit IgG antibody (goat) conjugated with HRP (Bio-rad laboratories; #1706515)
https://www.bio-rad.com/en-jp/sku/1706515-goat-anti-rabbit-igg-h-l-hrp-conjugate?ID=1706515

The anti-GFP polyclonal antibodies (Invitrogen; #A-6455)
https://www.thermofisher.com/antibody/product/GFP-Antibody-Polyclonal/A-6455

Dual use research of concern

Policy information about [dual use research of concern](#)

Hazards

Could the accidental, deliberate or reckless misuse of agents or technologies generated in the work, or the application of information presented in the manuscript, pose a threat to:

No	Yes	
☒	☐	Public health
☒	☐	National security
☒	☐	Crops and/or livestock
☒	☐	Ecosystems
☒	☐	Any other significant area

Experiments of concern

Does the work involve any of these experiments of concern:

No	Yes	
☒	☐	Demonstrate how to render a vaccine ineffective
☒	☐	Confer resistance to therapeutically useful antibiotics or antiviral agents
☒	☐	Enhance the virulence of a pathogen or render a nonpathogen virulent
☒	☐	Increase transmissibility of a pathogen
☒	☐	Alter the host range of a pathogen
☒	☐	Enable evasion of diagnostic/detection modalities
☒	☐	Enable the weaponization of a biological agent or toxin
☒	☐	Any other potentially harmful combination of experiments and agents

Plants

Seed stocks	Arabidopsis thaliana accession Columbia-0 (Col-0) was used as the wild-type. Mutants used in this study were wdr48-1 (GABI_585_E01; At3g05090), wdr48-2 (SALK_059570C; At3g05090), aha1-9 (SAIL_1285_D12; At2g18960), cbc1-1 (SALK_005187; At3g01490), cbc2-1 (SAIL_740_G01; At5g50000), phot1-5 phot2-1 (Kinoshita et al., 2001, Nature 414, 656–660), phot1-5 (Huala et al., 1997, Science 278, 2120–2128), phot2-1 (Kagawa et al., 2001, Science 291, 2138–2141), blus1-1, and phot1-GFP (Takemiya et al., 2013, Nat. Commun. 4, 2094).
Novel plant genotypes	WDR48pro:GFP-WDR48, WDR48pro:GFP-S393A, WDR48pro:GFP-S393E, and 35Spro:3xFLAG-WDR48 were introduced into the wdr48-1 background. GC1pro:OPE7-mCherry was introduced into phot1-GFP- or GFP-WDR48-expressing plants. Details of their generation are described in the Methods section. WDR48pro:GFP-S393E/phot1-5 phot2-1 wdr48-1, WDR48pro:GFP-S393E/blus1-1 wdr48-1 and WDR48pro:GFP-S393E/aha1-9 wdr48-1 were produced by crossing WDR48pro:GFP-S393E plants with phot1-5 phot2-1, blus1-1, or aha1-9 mutants.
Authentication	Homozygous T-DNA insertion mutants were isolated by genotyping PCR. Transgenic plants produced in this study were isolated by kanamycin, hygromycin, or FastR screening. Two independent lines were used for experiment to avoid potential side effects caused by T-DNA insertion.
